# Trends of Postoperative Radiotherapy for Completely Resected Non-small Cell Lung Cancer in China: A Hospital-Based Multicenter 10–Year (2005–2014) Retrospective Clinical Epidemiological Study

**DOI:** 10.3389/fonc.2019.00786

**Published:** 2019-08-20

**Authors:** Yu Men, Le Wang, Ye Zhang, Shugeng Gao, Junling Li, Ning Wu, Boyan Yang, Shangmei Liu, Jiansong Ren, Yunchao Huang, Debin Wang, Xianzhen Liao, Xiaojng Xing, Lingbin Du, Li Yang, Yuqin Liu, Yongzhen Zhang, Donghua Wei, Yunyong Liu, Kai Zhang, Youlin Qiao, Jufang Shi, Wanqing Chen, Min Dai, Zhouguang Hui

**Affiliations:** ^1^Department of VIP Medical Services & Radiation Oncology, National Cancer Center/National Clinical Research Center for Cancer/Cancer Hospital, Chinese Academy of Medical Sciences and Peking Union Medical College, Beijing, China; ^2^Office of Cancer Screening, National Cancer Center/National Clinical Research Center for Cancer/Cancer Hospital, Chinese Academy of Medical Sciences and Peking Union Medical College, Beijing, China; ^3^Institute of Cancer Research and Basic Medical Sciences of Chinese Academy of Sciences, Zhejiang Cancer Hospital, Cancer Hospital of University of Chinese Academy of Sciences, Hangzhou, China; ^4^Department of Radiation Oncology, National Cancer Center/National Clinical Research Center for Cancer/Cancer Hospital, Chinese Academy of Medical Sciences and Peking Union Medical College, Beijing, China; ^5^Department of Thoracic Surgery, National Cancer Center/National Clinical Research Center for Cancer/Cancer Hospital, Chinese Academy of Medical Sciences and Peking Union Medical College, Beijing, China; ^6^Department of Medical Oncology, National Cancer Center/National Clinical Research Center for Cancer/Cancer Hospital, Chinese Academy of Medical Sciences and Peking Union Medical College, Beijing, China; ^7^Department of Diagnostic Radiology, National Cancer Center/National Clinical Research Center for Cancer/Cancer Hospital, Chinese Academy of Medical Sciences and Peking Union Medical College, Beijing, China; ^8^Department of General Medicine, National Cancer Center/National Clinical Research Center for Cancer/Cancer Hospital, Chinese Academy of Medical Sciences and Peking Union Medical College, Beijing, China; ^9^Department of Pathology, National Cancer Center/National Clinical Research Center for Cancer/Cancer Hospital, Chinese Academy of Medical Sciences and Peking Union Medical College, Beijing, China; ^10^Yunnan Cancer Hospital, Kunming, China; ^11^School of Health Services Management, Anhui Medical University, Hefei, China; ^12^Hunan Office for Cancer Control and Research, Hunan Cancer Hospital, Changsha, China; ^13^Liaoning Office for Cancer Control and Research, Liaoning Cancer Hospital & Institute, Shenyang, China; ^14^School of Public Health, Guangxi Medical University, Nanning, China; ^15^Cancer Epidemiology Research Center, Gansu Provincial Cancer Hospital, Lanzhou, China; ^16^Department of Epidemiology, Shanxi Provincial Cancer Hospital, Taiyuan, China; ^17^Cancer Department of Physical Examination, Anhui Provincial Cancer Hospital, Hefei, China; ^18^Cancer Department of Physical Examination, National Cancer Center/National Clinical Research Center for Cancer/Cancer Hospital, Chinese Academy of Medical Sciences and Peking Union Medical College, Beijing, China; ^19^Department of Epidemiology, National Cancer Center/National Clinical Research Center for Cancer/Cancer Hospital, Chinese Academy of Medical Sciences and Peking Union Medical College, Beijing, China

**Keywords:** NSCLC, postoperative radiotherapy, trend, epidemiology, multicenter

## Abstract

**Objectives:** The role of postoperative radiotherapy (PORT) in the treatment of patients with completely resected non-small cell lung cancer (NSCLC) is not clear. Few study explored the trends of the PORT use. In this study, we examine the status of PORT use of completely resected NSCLC in mainland China.

**Methods:** From 2005 to 2014, patients with primary lung cancer from eight hospitals across seven geographic regions of mainland China were selected. Then patients with staged I–IIIA NSCLC receiving radical surgery were enrolled in this study. The chi-square test was used to compare differences in the use of PORT among the groups of different age, regions and stages. The Cochran-Armitage trend test was used to identify the trend in the PORT use from 2005 to 2014.

**Results:** Totally, 2,253 out of 7,184 patients were with staged I–IIIA NSCLC receiving completely resection. Only 122 patients (5.42%) received PORT. During this decade, the use of PORT declined significantly (*p* = 0.0002). In high socio-economic areas, the percentage of PORT use was 7.43%, which was significantly higher than 1.34% in the low socio-economic areas (*p* < 0.0001). Age was also associated with PORT use (*p* = 0.0747). For N0-1 and N2 NSCLC, the proportions of PORT use were 4.01 and 10.22%, respectively (*p* < 0.0001). And in N0-1 or N2 NSCLC, the proportions both decreased significantly during this decade (*p* = 0.009 and 0.026, respectively). For stage I, IIA, IIB and IIIA, the proportions who received PORT were 2.59, 4.65, 5.49, and 10.29%, respectively (*p* < 0.0001). Modern radiation techniques were widely used, but the volumes and doses varied widely. The proportions of using IMRT and EPID/IGRT increased after 2012.

**Conclusions:** In China, the use of PORT was less than developed countries and had a declined trend. The use of PORT was related to disease stages, patients' age and geographic location. Both in N0-1 and N2 diseases, the use of PORT declined. Proper education of radiation doctors was urgently needed.

## Introduction

Based on 2008 estimates, throughout the world, lung cancer accounts for 13% of the total cases of cancer and 18% of the cancer-related deaths (1). In China, lung cancer remains the most common incident cancer and the leading cause of cancer death (2).

Non-small cell lung cancer (NSCLC) accounts for 80–85% of all cases of lung cancer. Surgery remains the most important treatment for stage I/II and IIIA NSCLC. However, the local-regional recurrence is common, occurring in approximately 20% of patients with stage I disease and in up to 50% of patients with stage III disease (3–7). Postoperative radiotherapy (PORT) may reduce local-regional recurrence. However, a landmark meta-analysis study on PORT published in 1998 concluded that PORT was detrimental (HR 1.21, *p* = 0.001) (8). But many radiation oncologists remain skeptical about this results because of the toxicities, especially therapy-related deaths caused by suboptimal, outdated irradiation equipment and techniques, and the unacceptable radiation doses (8, 9). In recent years, the modern treatment techniques, 3D planning, linear accelerators have developed rapidly which could make radiotherapy more effective and less toxic (10–13). In 2004, chemotherapy became standard of care when the International Adjuvant Lung Cancer Trial (IALT) demonstrated that in comparison to surgery alone, cisplatin-based adjuvant therapy improved survival in patients with resected NSCLC (14). Given the above, the role of PORT has remained controversial for decades.

In the aspect of epidemiologic study about PORT in NSCLC, only one study examined the temporal trends based on Surveillance, Epidemiology, and End Results (SEER) Program published in 2006 (15). Up to now, few study examined the trends in the use of PORT especially in China. Besides, the emerging of new evidences of PORT and the development of radiation technologies may broaden the application of radiotherapy. Thus, we design this hospital-based multicenter retrospective clinical epidemiological study to illustrate the shift of PORT use of completely resected NSCLC in mainland China from 2005 to 2014.

## Methods

### Study Design

This study was a hospital-based multicenter 10 year (2005–2014) retrospective clinical epidemiological study of randomly selected primary lung cancer cases via medical chart review.

### Data Collection

Hospital selection, case sampling, and data collection methods have been previously described in detail (16). China was stratified into seven geographic regions (north, northeast, central, south, east, northwest, and southwest) according to the traditional administrative district definition. Eight hospitals from these seven regions were selected to provide cases. In each hospital, 1 month each year from 2005 to 2014 was randomly selected to review the entire cases except for January and February. According to the case report forms (CRF), well-trained clerks coded and categorized the selected data, and then send the data to National Office of CanSPUC for the data check.

The study protocol was approved by the Institutional Review Board of the Cancer Hospital of Chinese Academy of Medical Sciences.

### Patient Selection

Patients included in this study must meet the following inclusion criteria: (1) pathologically confirmed primary NSCLC, (2) patients must receive radical operation of lung cancer, staged I–IIIA, (3) inpatient admission date within the selected month in the study hospital, (4) patient characteristics and treatment (surgery, chemotherapy, or radiotherapy) were recorded.

Patient characteristics collected contained general information (age, sex, and smoking history), surgical approach, pathology, pathological stage, and the use of chemotherapy and radiotherapy. Pathological stage of cancer was categorized according to the seven edition of American Joint Committee on Cancer (AJCC) TNM System. The details of radiotherapy technology, target volume, dose, and the sequence of radiotherapy and chemotherapy were also collected.

### Statistical Analysis

The chi-square test was used to compare differences of the use of PORT among different age, region and stage groups. Then, independent factors were identified using Logistic regression analysis with 95% confidence interval (95% CI) for variables with a *p* < 0.05 in chi-square test. The Cochran-Armitage trend test was used to identify the trend in use of PORT from 2005 to 2014. Statistical significance was assessed by using 2-tailed tests with an alpha level of 0.05. SAS statistical software (version 9.4, SAS Institute Inc, Cary, NC) was used to analyze the data.

## Results

From 2005 to 2014, a total number of 7,184 lung cancer patients across the seven geographic regions were collected. Of these patients, 4,211 patients were pathologically diagnosed as NSCLC. After matching the aforementioned criteria, 2,253 patients with staged I–IIIA NSCLC receiving radical surgery were enrolled in this study.

Table 1 presents the characteristics of the enrolled patients. In general, most patients (75.2%) were ≤65 years old. The majority (70.7%) of the patients were male. More than half patients had a history of smoking. Squamous carcinoma and adenocarcinoma accounted for 91.1% of all patients. There were 927 (41.1%) patients with stage I disease, 733 (32.6%) with stage II, and 593 (26.3%) with stage III. Totally, 688 (30.5%) patients received chemotherapy.

**Table 1:** The characteristics of staged I–IIIA NSCLC patients received surgery.

	**All (*N* = 2,253)**	**PORT (*N* = 122)**	**Non-PORT (*N* = 2,131)**
	**No. (%)**	**No. (%)**	**No. (%)**
**Age, years**			
≤65	1694 (75.2)	100 (81.9)	1594 (74.8)
>65	559 (24.8)	22 (18.1)	537 (25.2)
**Sex**			
Male	1592 (70.7)	92 (75.4)	1500 (70.4)
Female	661 (29.3)	30 (24.6)	631 (29.6)
**Smoking history**			
Yes	1310 (58.2)	81 (66.4)	1229 (57.7)
No	909 (40.3)	40 (32.8)	869 (40.8)
Unknown	34 (1.5)	1 (0.8)	33 (1.5)
**Pathology**			
Squamous carcinoma	972 (43.2)	67 (54.9)	905 (42.5)
Adenocarcinoma	1079 (47.9)	47 (38.5)	1032 (48.4)
Adenosquamous carcinoma	86 (3.8)	2 (1.6)	84 (3.9)
Large cell carcinoma	21 (0.9)	0 (0.0)	21 (1.0)
Others	95 (4.2)	6 (4.9)	89 (4.2)
**Surgical method**			
Wedge Resection	59 (2.6)	3 (2.5)	56 (2.6)
Segmental Resection	55 (2.4)	3 (2.5)	52 (2.4)
Lobectomy	1911 (84.8)	98 (80.2)	1813 (85.1)
Pneumonectomy	119 (5.3)	9 (7.4)	110 (5.2)
Unknown	109 (4.9)	9 (7.4)	100 (4.7)
**Stage**			
I	927 (41.1)	24 (19.6)	903 (42.4)
IIA	387 (17.2)	18 (14.8)	369 (17.3)
IIB	346 (15.4)	19 (15.6)	327 (15.3)
IIIA	593 (26.3)	61 (50.0)	532 (25.0)
**Stage, T**			
T1	489 (21.7)	18 (14.8)	471 (22.1)
T2	1326 (58.9)	81 (66.4)	1245 (58.4)
T3	438 (19.4)	23 (18.9)	415 (19.5)
**Stage, N**			
N0	1327 (58.9)	41 (33.6)	1286 (60.3)
N1	417 (18.5)	29 (23.8)	388 (18.2)
N2	509 (22.6)	52 (42.6)	457 (21.5)
**Chemotherapy**			
No	1541 (68.4)	40 (32.8)	1501 (70.4)
Neoadjuvant	5 (0.2)	0 (0.0)	5 (0.2)
Adjuvant	683 (30.3)	80 (65.6)	603 (28.3)
Unknown	24 (1.1)	2 (1.6)	22 (1.1)
**Hospital**			
Hunan	229 (10.2)	3 (2.5)	226 (10.6)
Shanxi	383 (17.0)	28 (23.0)	355 (16.7)
Liaoning	804 (35.7)	75 (61.6)	729 (34.2)
Zhejiang	91 (4.0)	6 (4.9)	85 (4.0)
Yunan	314 (13.9)	0 (0.0)	314 (14.7)
Gansu	32 (1.4)	0 (0.0)	32 (1.5)
Anhui	283 (12.6)	5 (4.1)	278 (13.0)
Guangxi	117 (5.2)	5 (4.1)	112 (5.3)

**Figure 1:**
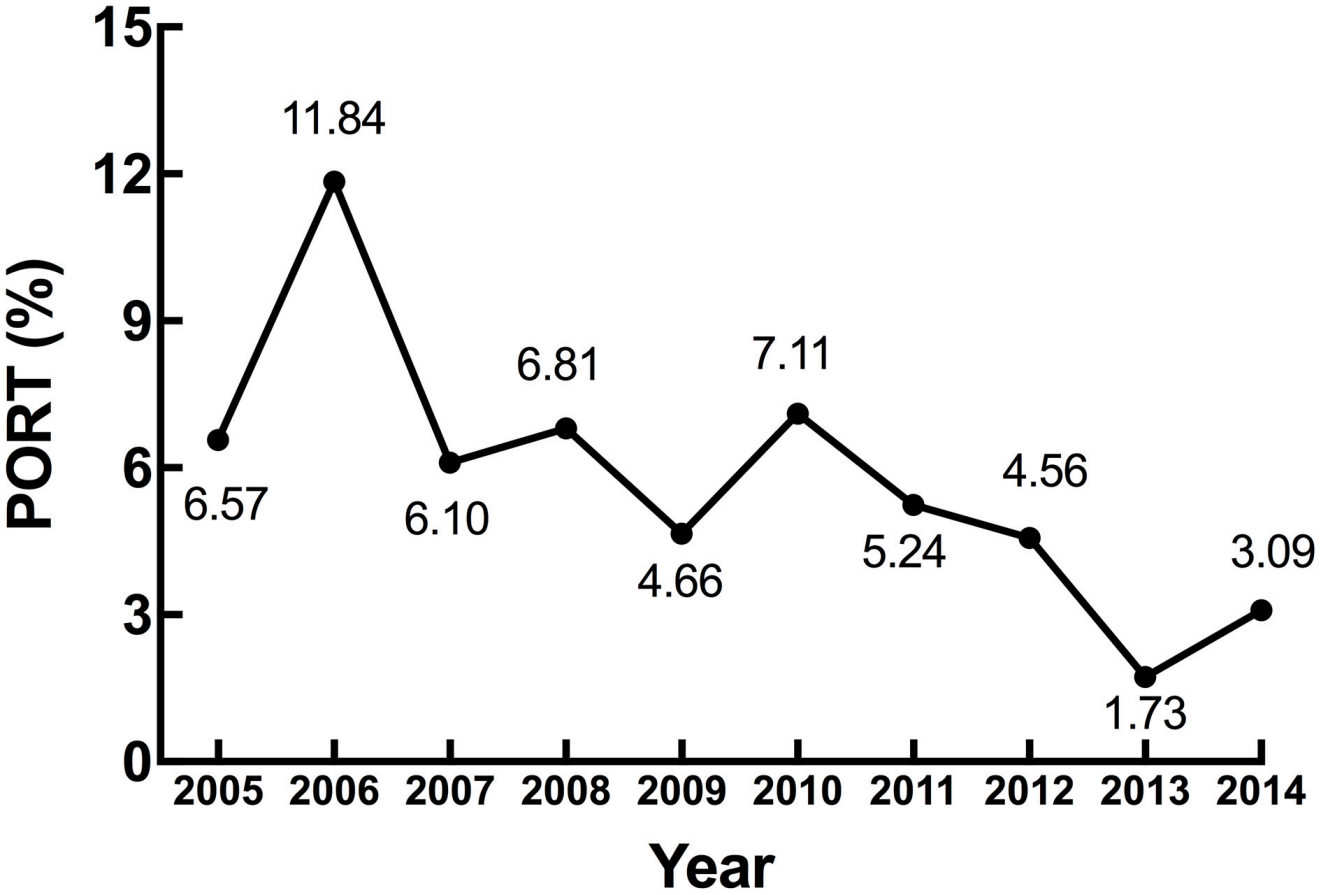
Trend of PORT use for patients from 2005 to 2014.

Overall, 122 patients received PORT, which occupied 5.42% of all the patients. From 2005 to 2014, the proportion of using PORT decreased significantly, from 6.57% in 2005 to 3.09% in 2014 (*p* = 0.0002). In 2013, the proportion was only 1.73% (Figure 1).

**Figure 2:**
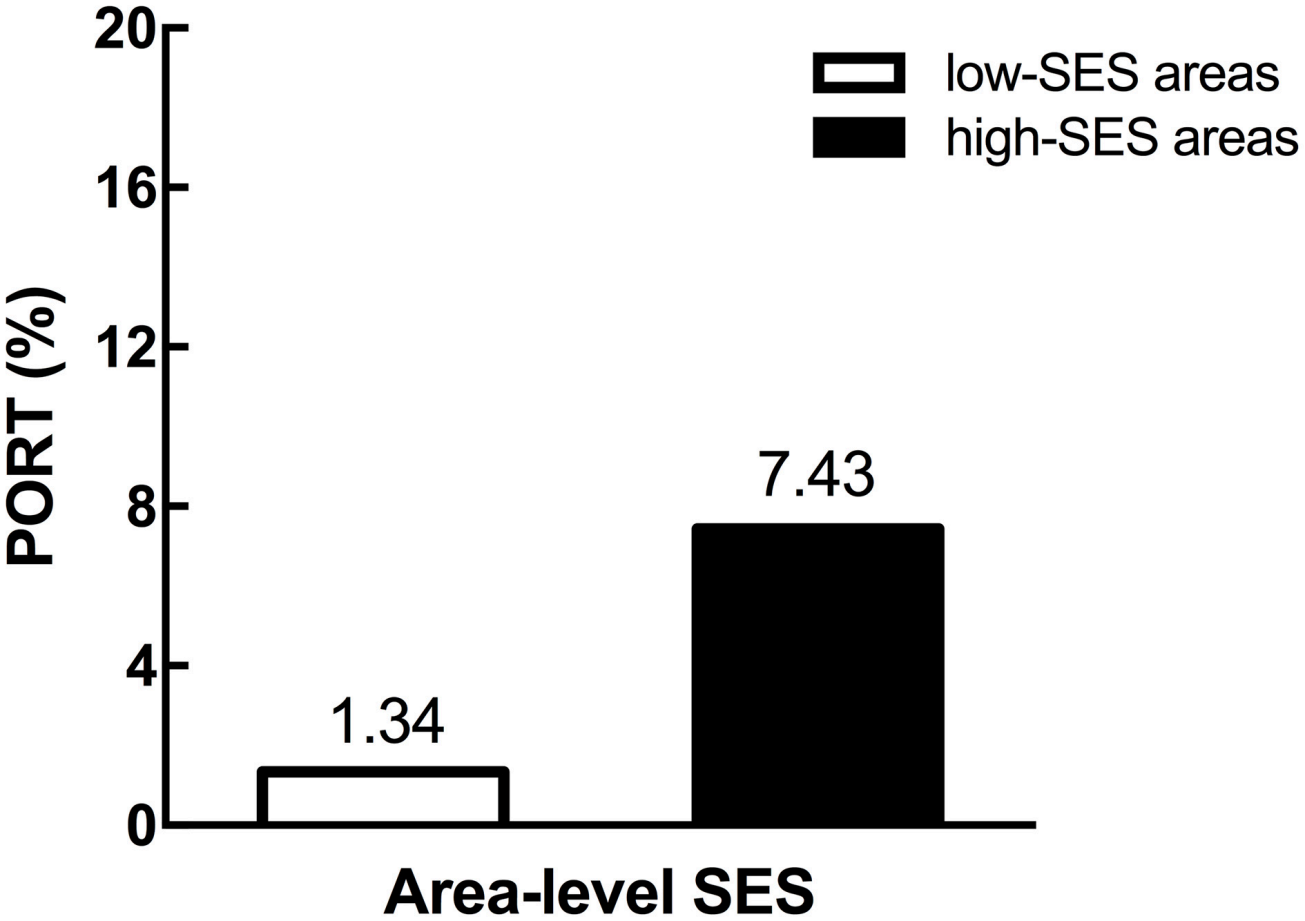
The proportion of PORT use according to area-level SES.

As in our previous study, we had measured the area-level socioeconomic status (SES) (16). According to all the indicator variables, the eight provinces were grouped into low- and high-SES areas. High-SES areas included Hunan, Shanxi, Liaoning, and Zhejiang provinces, while low-SES areas included Yunnan, Gansu, Anhui, and Guangxi provinces. As shown in Figure 2, the percentages of PORT use were 1.34% in low—SES areas and 7.43% in high-SES areas, respectively, and the difference was statistically significant (*p* < 0.0001).

**Figure 3:**
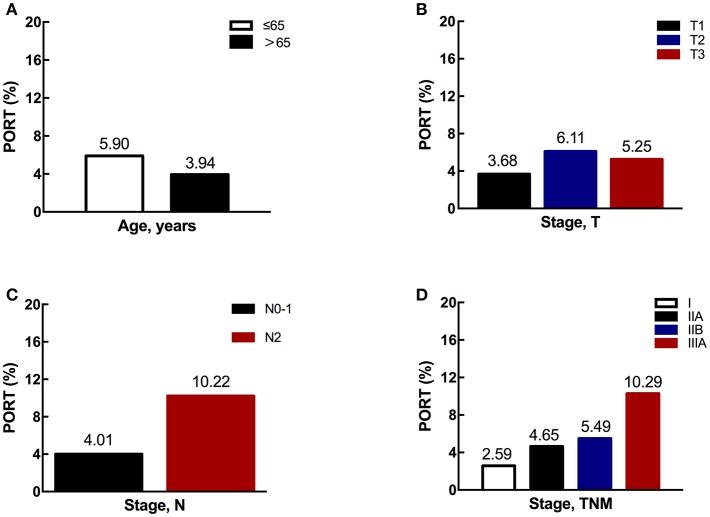
The proportions of PORT use according to different characteristics. **(A)** The proportion of PORT use according to age. **(B)** The proportion of PORT use according to T stage. **(C)** The proportion of PORT use according to N stage. **(D)** The proportion of PORT use according to TNM stage.

**Figure 4:**
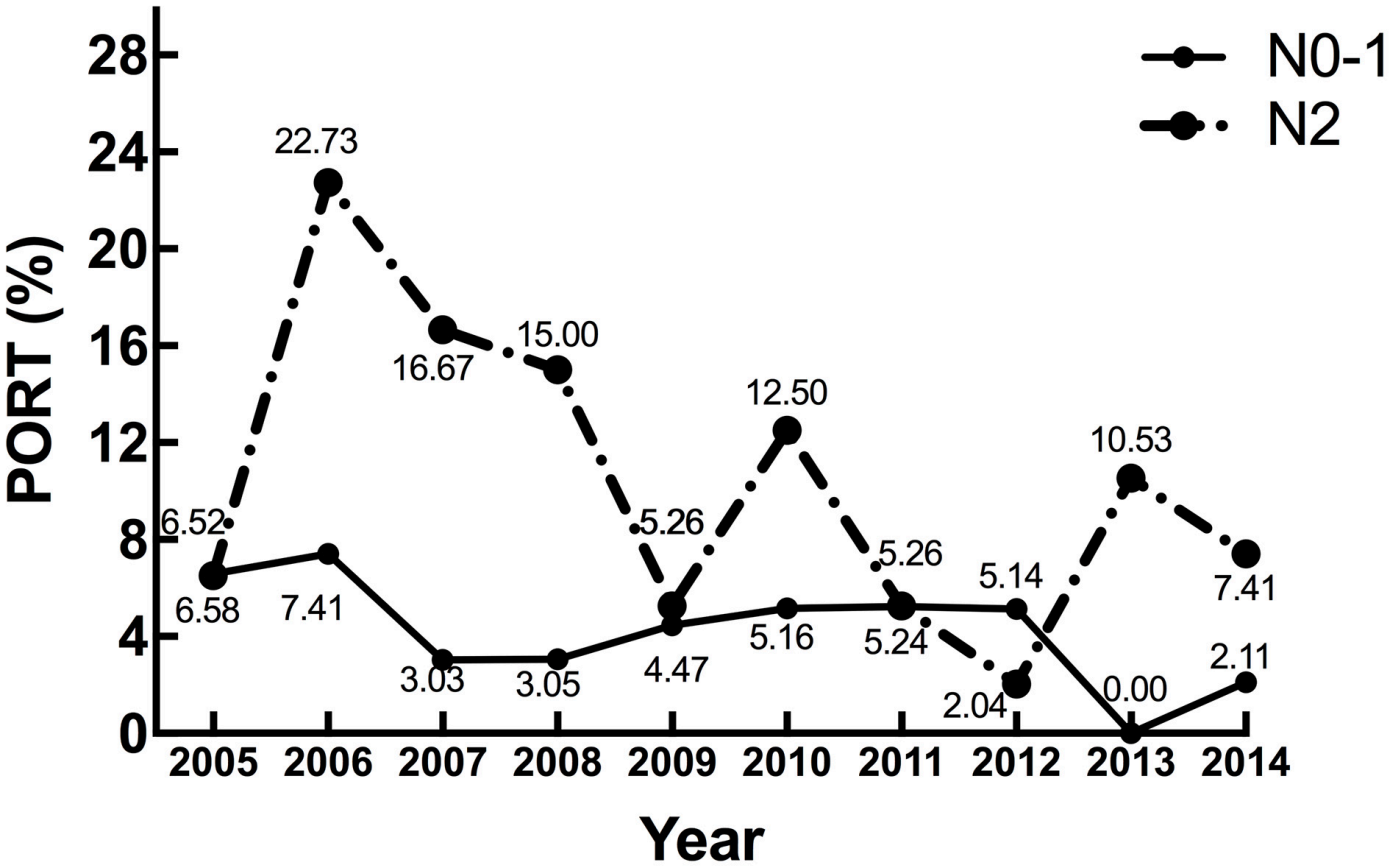
The trends of PORT use for different N stage patients from 2005 to 2014.

Next, we explored the association between different characteristics (including age and stage) and PORT use. Patients ≤65 years old had an overall PORT use rate of 5.90%, which dropped to 3.94% for those older than 65 years old (shown in Figure 3A). The difference was marginally significant (*p* = 0.0747). Additionally, there was no significant difference between PORT use and T stage (*p* = 0.1262, shown in Figure 3B). However, the PORT use elevated significantly with the increasing of N stage (*p* < 0.0001, shown in Figure 3C). The proportion of patients receiving PORT was 4.01% in N0-1 and 10.22% in N2, respectively. A similar trend was also found for TNM stage, with the proportion of 2.59, 4.65, 5.49, and 10.29% for stage I, IIA, IIB, and IIIA, respectively (*p* < 0.0001, shown in Figure 3D). When analyzing the trend of PORT use in different N stage patients during this decade, the proportions in N0-1 and N2 diseases both decreased significantly. For N0-1 diseases, the *p*-value was 0.009. For N2 diseases, the difference was significant (*p* = 0.026), and the proportion decreased especially after 2006 (Figure 4).

Multivariate analysis revealed that period, SES, N stage, and TNM stage, except age, were all independent factor for PORT use (Table 2).

**Table 2:** Multivariate analysis of independent factors for PORT use.

	**OR**	**95%CI**
**Period**		
2005–2009	1	–
2010–2014	0.606	0.419–0.874
**SES**		
High-SES	1	–
Low-SES	0.169	0.088–0.325
**Age**		
≤65	1	–
<65	0.653	0.407–1.047
**Stage, N**		
N0-1	1	–
N2	2.722	1.874–3.954
**Stage, TNM**		
I	1	–
IIA	1.835	0.984–3.421
IIB	2.186	1.182–4.043
IIIA	4.314	2.658–7.002

During the period of 2005 to 2014, modern radiation techniques had been widely used. In simulation, CT/4D-CT scans were commonly performed with a proportion of 68.0%. IMRT and 3D-CRT were applied in 80 patients (65.6%). In order to ensure the position accuracy, EPID and IGRT were recommended. But the survey illustrated the poorly utilization of these techniques (26.2%). The prescribed doses for PORT were delivered <45 Gy in 41 patients (33.6%), 45–54 Gy in 24 patients (19.7%) and >54 Gy in 45 patients (36.9%) (Table 3).

**Table 3:** The techniques and delivery of PORT.

	**No**.	**Percentage (%)**
**Simulation**		
X ray	31	25.4
CT^a^	81	66.4
4D-CT^b^	2	1.6
Unknown	8	6.6
**Planning**		
2D-RT^c^	39	31.9
3D-CRT^d^	69	56.6
IMRT^e^	11	9.0
Unknown	3	2.5
**Position verification system**		
No	8	6.6
EPID^f^	5	4.1
IGRT^g^	27	22.1
Unknown	82	67.2
**Target volume**		
Bronchial stump	45	36.9
Involved lymph node stations	19	15.6
Bronchial stump + Involved lymph node stations	2	1.6
Others	56	45.9
**Total Dose**		
<45 Gy	41	33.6
45–54 Gy	24	19.7
>54 Gy	45	36.9
Unknown	12	9.8

a CT, Computed Tomography.

b 4D-CT, 4-Dimensional Computed Tomography.

c 2D-RT, 2-Dimensional Radiation Therapy.

d 3D-CRT, 3-Dimensional Conformal Radiation Therapy.

e IMRT, Intensity-Modulated Radiation Therapy.

f EPID, Electronic Portal Imaging Device.

g IGRT, Image-Guided Radiation Therapy.

Then, we compared the differences of applied radiation techniques between 2005–2011 and 2012–2014. Simulation was performed using CT/4D-CT in most patients and with a stable application. The prescribed doses for PORT were also stable, but a little increase of prescription of 45–54 Gy could be observed. Importantly, we found the use of IMRT and position verification methods increased significantly (*p* = 0.014, 0.003, respectively) (Table 4).

**Table 4:** Changes of applied radiation techniques between 2005–2011 and 2012–2014.

	**2005–2011 (*****N*** **=** **97)**		**2012–2014 (*****N*** **=** **25)**		***p***
	**No**.	**Percentage (%)**	**No**.	**Percentage (%)**	
Simulation					0.172
X ray	22	22.7	9	36.0	
CT/4D-CT	67	69.1	16	64.0	
Unknown	8	8.2	0	0.0	
Planning					0.014
2D-RT	30	30.9	9	36.0	
3D-CRT	60	61.8	9	36.0	
IMRT	5	5.2	6	24.0	
Unknown	2	2.1	1	4.0	
Position verification system					0.003
No	6	6.2	2	8.0	
Yes	19	19.6	13	52.0	
Unknown	72	74.2	10	40.0	
Total Dose					0.546
<45 Gy	35	36.1	6	24.0	
45–54 Gy	17	17.5	7	28.0	
>54 Gy	36	37.1	9	36.0	
Unknown	9	9.3	3	12.0	

## Discussions

Our study is the first geographically representative epidemiologic study of PORT in NSCLC patients in China. The results showed a declined trend of PORT use from 2005 to 2014. The use of PORT was correlated with stage, especially N stage, rather than T stage. Moreover, patients' age and geographic location also affected the use of PORT. What's more, even in N2 NSCLC, as well as N0-1 NSCLC, the PORT use emerged a declined trend. PORT is the main potential treatment to further reduce local-regional recurrence of resected NSCLC. In the aspect of epidemiologic study, by now, only one study examined the temporal trends of PORT which based on Surveillance, Epidemiology, and End Results (SEER) Program. On the other hand, in the world cancer report published in 2014, it estimated that 35.8% of world's new lung cases would occur in China. And China as one of developing countries, in the health system, has many differences from the USA. Thus, it's necessary to design a survey to learn the Chinese epidemiological situation of the use of PORT in NSCLC.

Although PORT is a valuable treatment for NSCLC, it's not routinely recommended for all patients with resected NSCLC. The results of the 1998 meta-analysis showed that NSCLC patients receiving PORT were detrimental (17). In the USA, based on the research published in 2006, the use of PORT showed a declined trend (15). Similarly, based on our study, in mainland China the use of PORT for NSCLC also has substantially declined from 2005 to 2014. The TNM stage is the main factor affecting the choice of treatment, including PORT. Yet in the survey of USA, the relation between T or TNM stage with the use of PORT was not analyzed. As observed in our study, the use of PORT increased with the increase of TNM stage. When the T and N stages were considered separately, N stage was more important influencing the selection of PORT in NSCLC. Our results also showed that the T stage was not associated with the choosing of PORT. But with growing of the N stage, the proportions of PORT use were increased simultaneously.

In NCCN guideline and CSCO guideline, PORT is not recommended for patients with pathologic stage N0-1 disease, because it has been associated with increased mortality, at least when using older RT techniques. But PORT is preferred for N2 disease since it appears to improve survival significantly as an adjunct to postoperative chemotherapy in non-randomized analyses. A postoperative multimodality evaluation, including a consultation with a radiation oncologist, is recommended to assess benefits and risks of adjuvant radiotherapy in patients with N2 disease. Our survey showed that the trend of PORT in N0-1 diseases had declined, which was conformal with the recommendation in the aforementioned guidelines. In N2 diseases, the trend hypothetically should be increased or keep stable, but the realistic result was opposite. The possible reasons to explain the result may as follows: Firstly, although PORT is an option for N2-NSCLC patients, the evidence is far from enough. The benefit still needs to be confirmed by randomized clinical trials (RCTs). Up to now, there has been three such phase III RCTs: CALGB 9734 failed because of slow accrual; LUNGART and the other phase III multicenter RCT (NCT00880971) from our center are both ongoing now. Therefore, many radiation oncologists may remain skeptical about the use of PORT for N2 disease. Secondly, with the developing of medical insurance system in China, more NSCLC patients have the opportunity to receive surgery, which leads to the increase of pN2 population. Thirdly, resource shortage of radiation still exists in China. The comparison of the absolute value of the use of PORT for patients with N2 disease between China and America may also confirm this: in our study the proportion was only 10.22%, but in America even in 2002, it was 37% (15). Finally, it may be related to the lack of awareness and education about the standards of PORT and the excessive caution of side effects such as radiation induced pulmonary/cardiac toxicities.

Our study also showed the variation in PORT use with patient age and geographic location. Elder patients had less PORT than younger patients. In American study, they also demonstrated that age was highly predictive of PORT use. Despite of an effective treatment modality, PORT may cause a few adverse events including pulmonary and cardiac toxicities. Besides, after radical surgery, the tolerance of patient for radiation toxicities reduces. Patients older than 65 years usually have some complications which must take into consideration when choosing alternative treatments such as PORT. Thus, compared with younger ones, patients above 65 years old had less opportunity to undertake PORT, though it was not an independent factor. The area-based SES judging from seven indicators accurately reflected the multidimensional character of regional socioeconomic position. As a part of our results, the high-SES areas had a high percentage of PORT use. In the surveys conducted by Yin et al. (18), which investigated the changes of radiation oncology in mainland China, the equipment of radiotherapy had grown remarkably, and advanced techniques had been implemented very quickly from 1997 to 2011. But resources based on the population were still far less than the recommendation of the World Health Organization. Rural and remote areas were much less well-equipped which may due to financial problems. When diagnosed as malignant tumors, people living in mainland China traditionally seek for surgery and chemotherapy rather than radiotherapy, though well-educated patients may also consult radiation oncologists as well. Low-income populations often could not afford the high cost of radiotherapy. From above, we can explain that the PORT use is influenced by the economic strength and educational level.

In order to reduce potential pulmonary/cardiac toxic effects, PORT should be delivered with modern techniques such as CT-based 3D-CRT or IMRT planning, with which target volumes and normal tissue constraints can be precisely defined. In patients with locally advanced NSCLC treated with concurrent chemotherapy, IMRT significantly reduced the rate of high grade pneumonitis and improved higher overall survival compared to 3D-CRT (19). In our survey, we found that the simulation and planning of modern radiation treatment were widely used. When the techniques using before and after 2011 were compared, the use of IMRT was significantly increased and more position verification methods were applied, which implied the improvement of radiation in China. As recommended in NCCN guideline, the CTV includes the bronchial stump and high-risk draining lymph node stations. Previous studies showed that large variability was observed in routine target definition for PORT (20, 21). According to Lung ART study, routine CTVs varied up to 3-fold between clinicians (21). However, this variance was significantly reduced when clinicians were uniform trained. Therefore, the need for standardization must be emphasized. Total dose is another important issue of radiotherapy. Standard doses pointed in guidelines are 50 to 54 Gy in 1.8 to 2 Gy fractions. Corso et al. (22) found that PORT with doses of 45 to 54 Gy remained significantly associated with improved OS. Doses up to 54 Gy are improper unless having nodal extracapsular extension of microscopic positive margins. In our survey, although 45 to 54 Gy was prescribed more often after 2011, the doses in clinical practice seemed to be higher than the recommendation. About 37% patients received PORT dose more than 54 Gy.

There are some limitations in our survey. Firstly, all patients included in our study were ethnically Chinese. Secondly, selection bias may exist as we used the data from the leading public cancer hospital of the province which may not represent the whole population of the area. Thirdly, we used the convenience sampling instead of random sampling methods. Fourthly, the data quality is largely dependent on the clinician's documentation and the records on radiation of some patients were incomplete.

## Conclusions

In China from 2004 to 2015, the application of PORT, in both N0-1 and N2 diseases, was declined. Patients' age, geographic location, and disease stages were all affected the choice of PORT. Proper education of radiation oncologists was urgently needed. Although modern radiation techniques were gradually applied during the past years, the optimal radiation volume and dose should be emphasized.

## Ethics Statement

This retrospective study was approved by the Institutional Ethics Review Board at the Institutional Review Board of the Cancer Hospital and Institute of Chinese Academy of Medical Sciences & Peking Union Medical College. Informed consent was exempted by the board due to the retrospective nature of this research. Patient records were anonymized and de-identified prior to analysis.

## Author Contributions

JS, MD, and ZH: study concepts and design. YeZ, SG, JL, NW, BY, SL, and JR: CRF design and data management. YH, DWa, XL, XX, LD, LY, YuqL, YoZ, DWe, and YunL: data collection. YM and LW: data analysis and interpretation, statistical analysis, manuscript preparation, and manuscript editing. KZ, YQ, and WC: program supervision. YM, LW, JS, MD, and ZH: manuscript reviewing and approving. All authors read and approved the final manuscript.

### Conflict of Interest Statement

The authors declare that the research was conducted in the absence of any commercial or financial relationships that could be construed as a potential conflict of interest.
